# Impact of limb amputation and cisplatin chemotherapy on metastatic progression in mouse models of osteosarcoma

**DOI:** 10.1038/s41598-021-04018-9

**Published:** 2021-12-24

**Authors:** L. Ren, S. Huang, J. Beck, Amy K. LeBlanc

**Affiliations:** Comparative Oncology Program, Center for Cancer Research, National Cancer Institute, National Institutes of Health, 37 Convent Drive, Room 2144, Bethesda, MD 20892 USA

**Keywords:** Cancer models, Cancer, Metastasis

## Abstract

Development of animal models that accurately recapitulate human cancer is an ongoing challenge. This is particularly relevant in the study of osteosarcoma (OS), a highly malignant bone tumor diagnosed in approximately 1000 pediatric/adolescent patients each year. Metastasis is the leading cause of patient death underscoring the need for relevant animal models of metastatic OS. In this study, we describe how existing OS mouse models can be interrogated in a time-course context to determine the kinetics of spontaneous metastasis from an orthotopically implanted primary tumor. We evaluated four highly metastatic OS cell lines (3 human, 1 mouse) to establish a timeline for metastatic progression in immune deficient NSG mice. To discern the effects of therapy on tumor development and metastasis in these models, we investigated cisplatin therapy and surgical limb amputation at early and late timepoints. These data help define the appropriate observational periods for studies of metastatic progression in OS and further our understanding of existing mouse models. Efforts to advance the study of metastatic OS are critical for facilitating the identification of novel therapeutics and for improving patient survival.

## Introduction

Osteosarcoma (OS), although a relatively uncommon malignancy, is the most common primary tumor of bone and the second highest cause of cancer-related death in pediatric patients. Children and adolescents are most commonly affected, with the peak incidence corresponding to the period of rapid skeletal growth^1^. Lung metastasis is clinically detectable at initial presentation in approximately 15–20% of patients^2^. For patients in which lung metastases are not detected, at least 30% will go on to develop metastases despite aggressive combination chemotherapy and surgery^3^. Survival for patients with metastatic or relapsed osteosarcoma is poor, with 5-year survival rates of less than 25%^4^.

The use of mouse models in cancer research is an important part of basic and translational cancer research^2^. Mouse models of OS can provide greater insight into the complex mechanisms that underlie the pathogenesis and metastatic progression of this aggressive tumor. Moreover, there is potential to test and observe the in vivo response to various therapeutic strategies, such as chemotherapy and surgical intervention, not only at the primary tumor site, but also in common sites of metastases.

Methods used by researchers to interrogate metastatic progression in OS can be broadly termed as “spontaneous metastasis” and “experimental metastasis”^5–7^. The experimental metastasis model uses tail vein injection techniques to evaluate the capacity of cancer cells to extravasate from the systemic vasculature, arrest within the pulmonary capillary bed, and successfully establish new growth in the pulmonary parenchyma. The advantages of this model are the efficiency and high rate of lung metastasis, but clear drawbacks include the inability to replicate the metastatic cascade in its entirety and the synchronized introduction of numerous circulating tumor cells which is not representative of metastasis in clinical disease. Tumor cells can also be injected into the tibia; however, this model is no longer considered spontaneous because tumor cells can be directly seeded into the vasculature during implantation^8^. In the spontaneous metastasis model, orthotopic transplantation of cancer cells or tissues along the tibia leads to the development of a primary tumor with subsequent spread of tumor cells to secondary sites including the lung. This makes the spontaneous metastasis model less efficient, increases the time for metastases to develop, and requires surgical amputation to remove the primary tumor from the tumor-bearing mice under study. However, the clear advantage of this model is that the metastatic spread of tumor cells more closely resembles clinical disease in patients allowing the entire metastatic cascade to be monitored and modeled^9^. This is critical in a disease such as OS where metastasis continues to be the primary cause of cancer-related death^4,10^.

**Figure 1:**
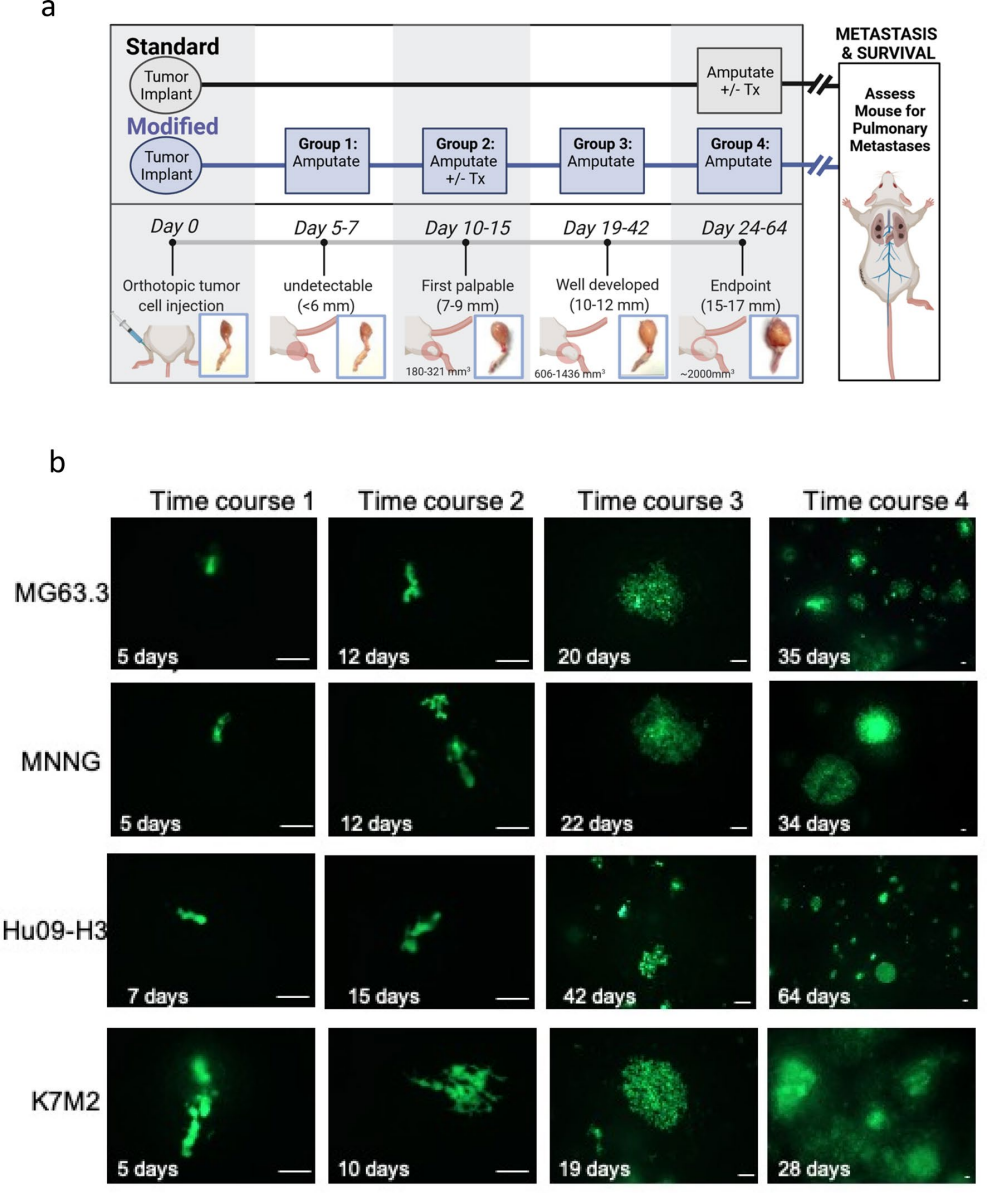
Primary tumors and lung metastases in OS cell bearing NSG mice. (**a**) Illustration comparing the standard surgical limb amputation protocol to our modified early amputation time course experiments including indication of the limb diameter and primary tumor size at each of the experimental time points. Additional information for each cell line is included in Table 1. (**b**) Representative images of GFP-tagged tumor cells (MG63.3, MNNG, Hu09-H3, K7M2) within the lung parenchyma at each limb amputation timepoint (time course 1–4). The freshly extracted mouse lungs were imaged without any additional processing. The images were taken at different magnification depending on the sizes of lung metastases. Scale bar = 100 µm.

In recent years, we have collected and thoroughly characterized ~ 20 human, murine and canine OS cell lines^11^ and employed highly metastatic cell lines for therapeutic drug testing on both primary tumor growth and metastatic progression using both experimental and spontaneous modelling approaches^12–14^. The spontaneous metastasis model is used widely for various applications in osteosarcoma research^15–19^. To date, these studies have focused on the implantation or injection of OS tumor tissues and cells within an orthotopic location, with limb amputations conducted when the primary tumors have reached 2000 mm^3^ in volume. When the spontaneous metastasis model is used in this way to evaluate the impact of drug treatment, we and others have observed that drugs which reduce metastasis also commonly inhibit primary tumor growth^12–14,20^. Therefore, it is difficult to discern whether inhibition of OS metastasis is due to the drug effect on primary tumor growth or directly on the process of metastatic progression. Ideally, one would conduct drug treatment experiments in a way where there would be minimal to no influence of primary tumor size and growth kinetics on the evaluation of metastasis. Therefore, to decipher the role of therapy on metastatic progression while minimizing the influence of the primary tumor growth, we conducted a series of experiments in which primary tumors were resected at different stages within immune deficient NSG (NOD *scid* gamma, The Jackson Laboratory, strain code 005557) mice. We hypothesized that metastatic tumor cells invade the lung early after orthotopic implantation, and that modifications to the standard amputation protocol will optimize the study of micrometastases while eliminating further contributions from the primary tumor. Tumors were derived from highly metastatic human (MNNG, MG63.3, Hu09-H3) and murine (K7M2) OS cell lines. Critically, similar survival was observed regardless of whether the primary tumors were resected early (200–300 mm^3^) or later in development (~ 2000 mm^3^), indicating that primary tumors can be removed at a very early stage of growth without influencing metastatic progression. Based on this finding, we adjusted the spontaneous metastasis experimental protocol to include early tumor resection (200–300 mm^3^) allowing us to test the widely used anti-OS therapeutic drug, cisplatin, on metastatic progression alone. This modified model (schema depicted in Fig. 1a) better recapitulates human disease in which approximately one third of patients without detectable lung masses progress to metastatic disease after resection of their primary tumor and use of concurrent chemotherapy^3^. These data enable improved application of these existing model systems for scientific questions that are focused on the biology of metastatic progression and investigation of novel approaches to pharmacologically target OS metastasis.

## Materials and methods

### Cells

The murine OS cell line K7M2 was developed in our lab^21^. Human OS cell line HOS/MNNG was purchased from ATCC. Hu09-H3 was obtained from Dr. Jun Yokota (National Cancer Center Research Institute, Tokyo, Japan). MG63.3 cells were derived from MG63.2^21^ by a process of experimental metastasis^11,21^. All the cells were cultured in DMEM (Invitrogen, Carlsbad, CA) medium supplement with 10% fetal bovine serum (FBS) and glutamine. All cell lines were transfected with lentiviral green fluorescent protein (GFP) constructs (pSICO-eGFP or p960-X1-685-eGFP), with strong GFP expressing cells selected and subsequently enriched by fluorescent cell sorting as described previously^22^.

### In vivo tumor formation and amputation

Two million OS cells in 100 ul HBSS were injected orthotopically to the para-osseous region of the proximal tibia of 5–6 week old female NSG mice as previously described^21^. Briefly, tumor cells were suspended in phenol free HBSS, assessed for > 90% viability (trypan blue), and injected into the caudal gastrocnemius muscle body adjacent to the tibia^11–13^. The tumor bearing legs were measured weekly. The volume of orthotopic tumor was calculated as previously reported^23^. Tumors were measured with calipers to estimate tumor size based on two-dimensional cross-sectional measurements. Tumor volume was determined using the equation (D × d2)/6 × 3.12 (where D = the maximum diameter and d = the minimum diameter).

Tumor bearing limbs were resected at 4 time points according to the experimental design (Fig. 1a, Table 1). All animal experiments were performed under a National Cancer Institute Animal Care and Use Committee (IACUC)-approved animal study protocol (PB-083–3) in accordance with ARRIVE guidelines.

**Table 1:** Summary of experimental outcomes for OS cell lines (human: MG63.3, MNNG, Hu09-H3; mouse K7M2) for time course experiments designed to determine the impact of timing of surgical limb amputation and survival due to metastatic progression.

Time course		MG63.3	MNNG	Hu09-H3	K7M2
1	Amputation days after tumor cell injection	5	5	7	5
	Primary tumor size	Undetectable	Undetectable	Undetectable	Undetectable
	Median survival time (Days)	65	Undefined	Undefined	94
2	Amputation days after tumor cell injection	12	12	15	10
	Primary tumor size (mm^3^)	321 ± 60	230 ± 18	180 ± 41	268 ± 53
	Median survival time (Days)	60	62	226	77
3	Amputation days after tumor cell injection	20	22	42	19
	Primary tumor size (mm^3^)	796 ± 108	606 ± 91	697 ± 207	1436 ± 159
	Median survival time (Days)	59	62	126	74
4	Amputation days after tumor cell injection	35	34	64	24
	Primary tumor size (mm^3^)	2160 ± 433	2144 ± 427	1949 ± 402	2351 ± 701
	Median survival time (Days)	63	58	119	77

### Early pulmonary metastasis assessment

At each limb amputation time point, a subset of mice (n = 3) was sacrificed for assessment of pulmonary metastatic burden. The remainder (n = 7) were followed until experimental endpoint which varied based on tumor cell line (Table 1). The lungs were extracted and imaged with no further processing using Leica DM IRB inverted fluorescent microscope with an attached CCD camera.

### In vivo treatment with cisplatin

NSG mice (n = 46) were injected with 2 million MG63.3 cells, and then randomly divided into 4 groups. Group 1 and 2 underwent early amputation when tumor diameter reached 7–9 mm, and group 3 and 4 underwent late amputation when tumor diameter reached 15–17 mm. All mice were euthanized using CO_2_ on Day 60 for lung metastasis burden evaluation. In mice assigned to time course groups 2 and 4, cisplatin (West-Ward, 4 mg/kg; IP) was administered once a week starting on Day 15 after tumor cell injection. For a 20 g mouse, drug volume was adjusted to 0.1 ml using 0.9% sodium chloride. Control mice received a similar injection containing 0.1 ml sodium chloride (group 1 and 3). In consideration of cisplatin toxicity, the schedule was deployed as two-weeks on and one-week off. Mice were weighed once weekly to determine changes in body weight and were monitored regularly according to humane and experimental endpoints outlined in the IACUC protocol. All the methods were performed in accordance with the relevant guidelines and regulations.

### Fluorescence image and analysis

Leica MZ FLIII fluorescence stereo microscope and Leica DM IRB inverted research microscope were used to acquire lung metastasis images using Leica Application Suite X 3.6.0.20104 and Openlab 5.5.2 software. Fluorescence was quantified using Fiji (ImageJ 1.53c).

Statistical tests were performed in GraphPad Prism 8 version 8.4.3. The Mann–Whitney U test was used for nonparametric comparisons, and logrank (Mantel-Cox) with Bonferroni correction was used for comparisons of survival curves.

## Results

### Primary tumor growth and lung metastasis development at different limb amputation points

In this study, we evaluated four highly metastatic OS cell lines: three human cell lines (MG63.3, HOS-MNNG and Hu09-H3) and one murine cell line (K7M2). For each cell line, two million OS cells were orthotopically injected in the para-osseous aspect of the proximal tibia in 40 NSG mice. Following injection, mice were randomly divided into 4 groups (each group n = 10) to assess different amputation time points. Each time point was selected based on the diameter of the tumor bearing leg (Fig. 1a; Table 1). Since OS cell lines develop primary tumors at different rates, the amputation time points varied slightly by cell line and included the time point at which the primary tumors are visibly undetectable (diameter of the tumor bearing leg < 6 mm), first palpable (diameter of the tumor bearing leg 7–9 mm), well-developed (diameter of the tumor bearing leg 10–12 mm), and at the experimental endpoint (~ 2000 mm^3^ tumor volume or diameter of the tumor bearing leg 15–17 mm). At each time point, 7 out of 10 mice from each group had limb amputation surgeries, and the remaining 3 mice were sacrificed to allow examination of the lungs under fluorescent microscopy.

At the first time point, a small number of GFP-tagged tumor cells are visible in the lungs even though the primary tumors are visibly undetectable (Fig. 1a). At this stage, very few (< 10) cells are observed in the lungs of several mice. The metastatic cells appeared as 1–2 cells clusters and are elongated with cytoplasmic protrusions (Fig. 1b, time course 1).

At the second time point, the primary tumors have developed into palpable masses with diameters of tumor bearing legs measuring 7–9 mm (Fig. 1a). At this second time point, increased numbers of metastatic cells are identified in the lungs (Fig. 1b, time course 2) with several clusters of 1–5 cells observed. The mice bearing K7M2 tumors had the highest burden of metastatic cells (Fig. 1b, K7M2 time course 2) with a few clusters containing > 100 tumor cells.

At the third time point, the tumor bearing legs measured approximately 10–12 mm in diameter. In the lungs of these mice, many colonies (> 100 cells) of metastatic tumor cells are observed. In addition, many smaller tumor cell clusters (≤ 5 cells) are scattered throughout the lung parenchyma. In the lungs of mice with K7M2 tumors, widespread metastases were too numerous to count and included many, approximately 1 mm diameter, microscopic metastases (Fig. 1b, time course 3).

At the last time point, the primary tumors reached ~ 2000 mm^3^ and tumor bearing legs are 15–17 mm in diameter. As shown in Table 1, the mice bearing xenograft tumors of different cell lines reached this point with varying time lengths. This included a time course as short as 24 days (K7M2) or as long as 64 days (Hu09-H3). At this stage, the development of lung metastases also varies. Metastases from K7M2 cells were diffusely distributed throughout the lungs and included numerous small metastatic cell clusters and larger metastases (> 1 mm) visible by stereomicroscopy (Fig. 1b, time course 4). MG63.3, MNNG and Hu09-H3 also developed lung metastases with a similar diffuse pattern but with fewer metastases compared to K7M2 (Fig. 1b, time course 4). In addition to the lung, MNNG cells formed grossly evident metastases in the liver and axillary lymph nodes (data not shown).

### Survival assessment with primary tumor removal at variable stages of tumor development

To determine whether early surgical removal of the primary OS tumors reduce metastasis and extends survival, all mice which underwent limb amputation were followed to the experiment endpoint, which occurred at 105–250 days after tumor cell injection. Each mouse was carefully examined grossly for metastasis to internal organs.

**Figure 2:**
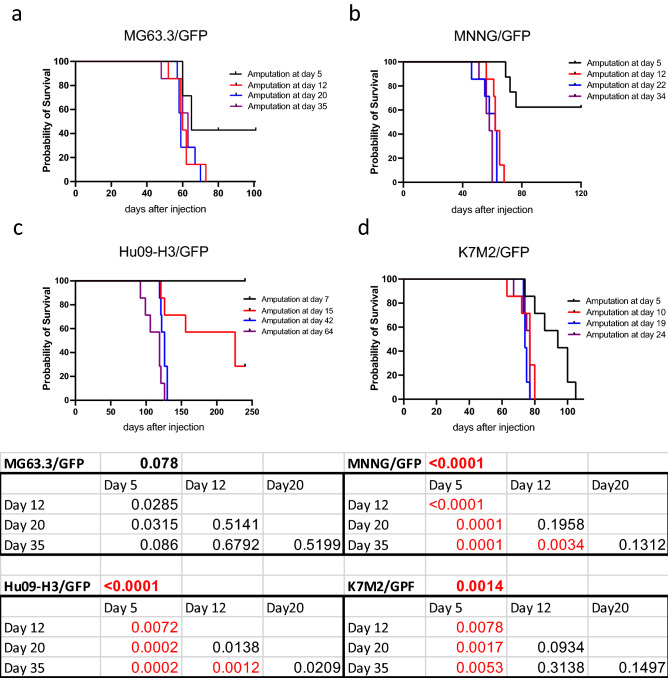
Metastatic outcomes of NSG mice bearing OS cell line xenografts. (**a**–**d**) Kaplan–Meier survival curves for mice based on timing of amputation following injection of MG63.3 (**a**), MNNG (**b**), Hu09-H3 (**c**), K7M2 (**d**). (**e**) Mantel–Cox identified significant differences in the survival curves of mice bearing MNNG, Hu09-H3, and K7M2 xenografts. Comparisons by amputation time point are included in the underlying tables. For multiple comparisons between timepoints within each cell line, a Bonferroni-corrected threshold for significance of < 0.0083 was used.

Survival curves for mice bearing MG63.3 xenografts are shown in Fig. 2a. All mice died with lung metastasis. Surprisingly, median survival times were generally between 59–65 days and did not vary based on the timing of surgery (Table 1). If amputation was performed at Day 5, when the tumors are not yet palpable, 3 out of 7 mice survived to the end of the experiment (101 days) and all surviving mice were free of detectable fluorescent metastatic cells or histologic evidence of metastasis within their lungs. A similar outcome was obtained from the experiment with MNNG cells (Fig. 2b). Four out of 7 mice which had leg amputation at Day 5 survived at the end of experiment (120 days) and were free of detectable metastases. Apart from the earliest amputation time point (5 days), all other mice succumbed to metastatic progression at around Day 60 regardless of whether limb amputation of the MNNG-derived primary tumor occurred at 12, 22 or 34 days. In addition to the lung, MNNG tumor cells also metastasized to the liver and axillary lymph nodes of all mice.

The mice bearing Hu09-H3 cells had relatively slow primary tumor growth and metastatic progression (Fig. 2c, Table 1). For these mice, early amputation (Day 7) resulted in a long survival, with all mice still alive when the experiment was terminated (250 days). In group 2 (limb amputation on Day 15), two out of 7 mice were still alive at the end of the experiment (250 days), but their survival time was widely variable (121–226 days). At the later timepoints of amputation (Days 42 and 64), all mice demonstrated a consistent median survival time (~ 120 days) (Fig. 2c) and all had a measurable burden of lung metastases.

For the most aggressive cell line, K7M2, although the amputations were performed before tumors were palpable (5 days), all mice still developed lung metastasis. However, mice amputated at Day 5 had a delayed progression of metastasis compared to mice which underwent amputation at later timepoints. Similar to mice bearing MNNG and Hu09-H3 xenografts, amputation at a very early stage of primary tumor development inhibited the development of life-limiting metastatic progression. Past this early timepoint, removal of the primary tumors via limb amputation did not have a significant impact on overall survival (Fig. 2d).

### Assessment of cisplatin treatment on OS metastasis

In our amputation model studies, we were able to characterize the behavior of multiple OS model systems in the context of limb amputation at various timepoints and stages of tumor development. In addition to primary tumor removal, human OS patients receive a multiagent chemotherapy protocol which includes cisplatin^24^. To determine whether limb amputation and chemotherapy exert a synergistic effect in these models, we next investigated the impact of cisplatin therapy on the development of metastases in mice bearing MG63.3 tumors undergoing early vs late amputation (design shown in Fig. 1a).

**Figure 3:**
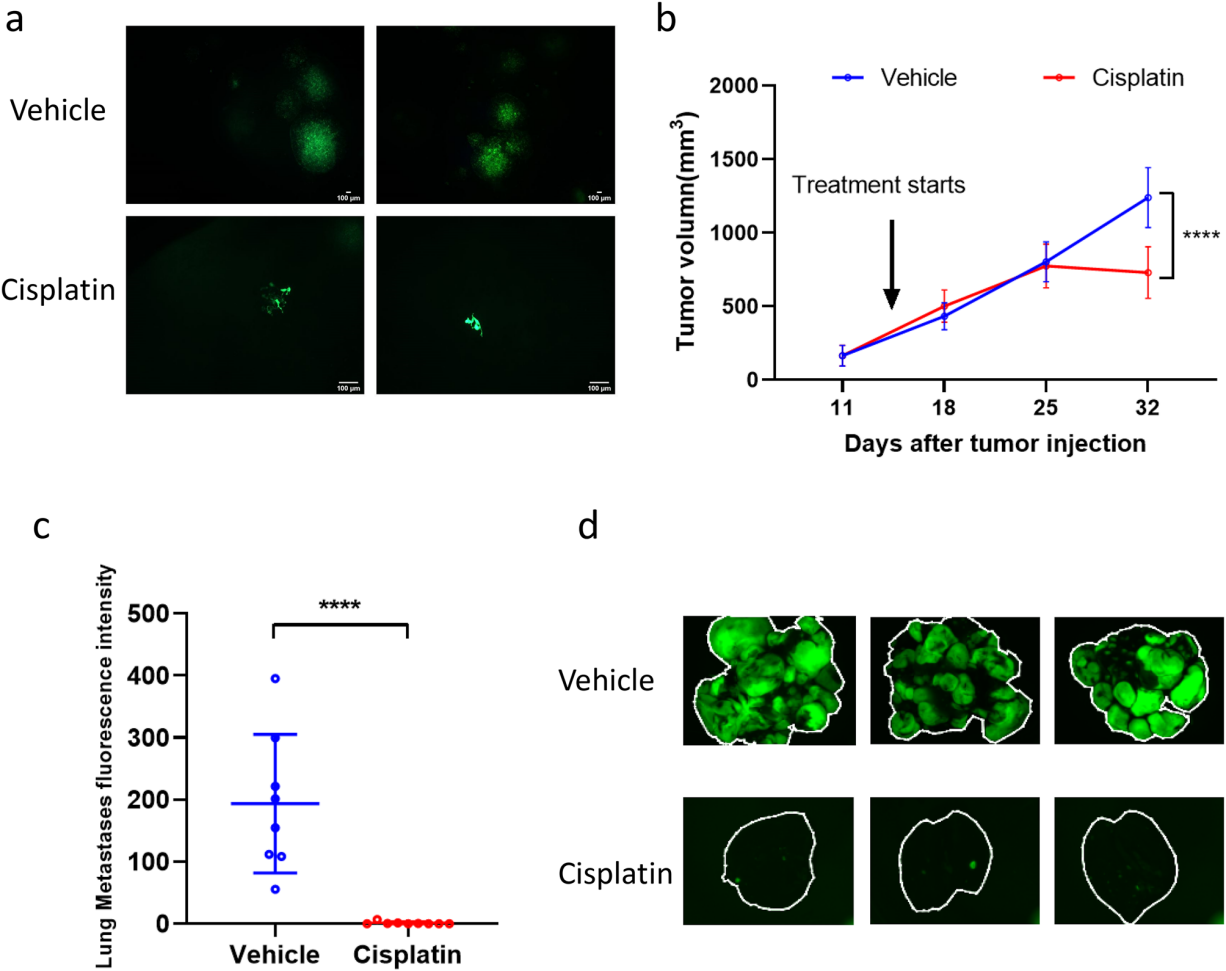
After late hindlimb amputation, both primary tumor and lung metastases of MG63.3 tumor cells were inhibited by cisplatin treatment. (**a**) Representative fluorescent images of lungs from mice that received vehicle or cisplatin treatment on day 35 following injection of MG63.3 tumor cells. Scale bar = 100 µm. (**b**) Graphical representation of primary tumor growth in mice receiving vehicle or cisplatin treatment. ****p < 0.0001. (**c**) Lung metastasis burden quantification at endpoint (Day 60). ****p < 0.0001. (**d**) Representative fluorescence images of pulmonary metastases (whole lung mount) at endpoint (Day 60).

First, we evaluated cisplatin treatment administered with a later timepoint of amputation when the primary tumor reaches the experimental endpoint, i.e., tumor bearing legs are 15–17 mm diameter, which is consistent with most investigators’ approach to a spontaneous model of metastasis. In this approach, cisplatin treatment was started when primary tumors were palpable (200–300 mm^3^). When primary tumors bearing legs reached 15–17 mm (Day 35), three mice from control and treated groups were randomly selected and euthanized to assess for the development of pulmonary metastases. In the control group, colonies of metastatic tumor cells were found throughout the lung and included numerous micro-metastases (< 10 cells) and large aggregates of > 1000 tumor cells (Fig. 3a). In contrast, less than 10 metastases were observed in cisplatin-treated mice, with most colonies comprised of a few cells, rarely over 100 (Fig. 3a). The remainder of mice in this experiment (8 in control group and 9 in cisplatin group) underwent amputation of the tumor-bearing limb on Day 35 at which time all mice had received cisplatin weekly for two weeks (one cycle of two-weeks-on, one-week-off treatment). The primary tumor volume of the cisplatin-treated group was significantly decreased compared to control (Fig. 3b). At the end of these experiments (Day 60), the lung metastasis burden of control group was also significantly lower in the cisplatin-treated mice (Fig. 3c,d).

**Figure 4:**
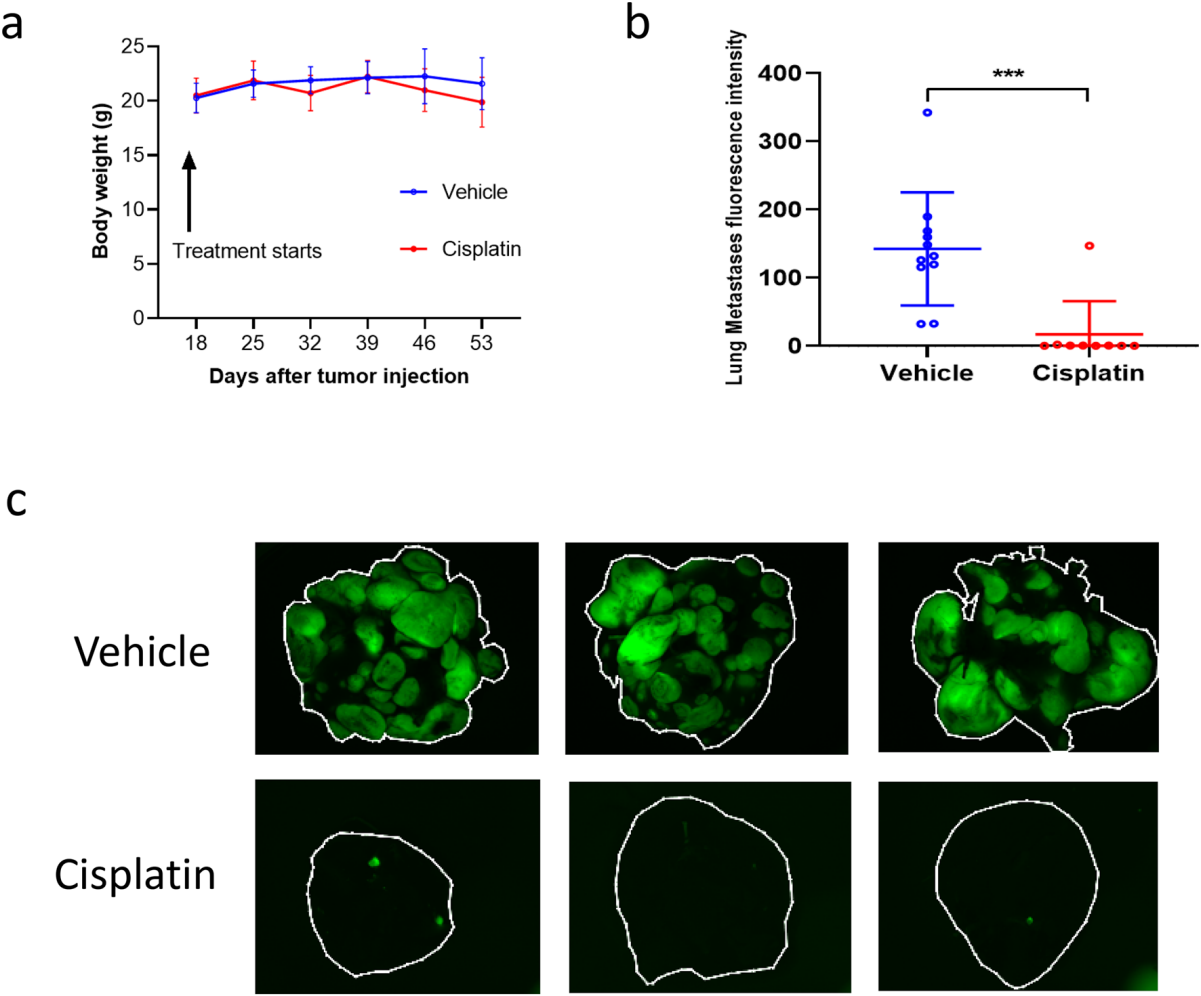
After early hindlimb amputation, cisplatin significantly inhibits MG63.3 tumor cell metastasis. (**a**) Body weight comparisons between treated and untreated mice through experiment. (**b**) Lung metastasis burden quantification at endpoint (Day 60). ***p = 0.0004. (**c**) Representative fluorescence images of pulmonary metastases (whole lung mount) at endpoint (Day 60).

After identifying a benefit of cisplatin in primary and metastatic tumor growth at late amputation time points, we next investigated the impact of cisplatin in mice undergoing early amputation. This allows for the investigation of drug treatment on very early-stage metastatic cells without the influence of the primary tumor growth. In this experiment, the limb amputations were performed when the primary tumor bearing legs were approximately 7–9 mm in diameter (Group 2, Fig. 1a). On the same day of amputation, three mice were randomly selected, sacrificed, and examined for the presence of lung metastases. Less than 5 colonies of 1–2 cancer cells were observed in each lung, confirming that metastatic cells had already reached and established in the lung, in a very similar context as presented in time course 2 in previous experiments (Fig. 1b). Cisplatin treatment was initiated three days after the limb amputation (11 control mice, 9 cisplatin-treated mice). Each mouse received a total of 4 doses, either vehicle or cisplatin. The experiment was terminated at Day 60 and lung metastases were evaluated with fluorescent microscopy. As shown in Fig. 4b,c, cisplatin therapy significantly suppressed metastatic progression in all but one mouse. In both early and late amputation settings, cisplatin therapy appeared well-tolerated, with no significant impact on body weight (Fig. 4a). Thus, cisplatin appears equally and highly effective in the treatment of lung metastasis in the MG63.3 model of OS, regardless of timing of limb amputation in the spontaneous model system.

## Discussion

The spontaneous metastasis mouse model is widely used for the study of metastasis biology in OS^5–7^. In this model, tumor cells are injected orthotopically within the soft tissue along the proximal tibia. Limb amputation is typically performed when the mouse primary tumor-bearing leg reaches 15–17 mm in diameter. When therapeutic drugs are tested in this model, the treatment usually starts when the primary tumor is just palpable and is continued as the primary tumor progresses to endpoint. Therefore, it is difficult to distinguish if the drug effect on metastasis is direct (i.e. cytotoxic effect specifically to metastatic cells) or is indirect (cytotoxic effect slowing the growth of the primary tumor, and in turn, reducing the magnitude of metastatic escape). In order to specifically observe the drug effect on metastasis and to minimize the influence of the primary tumor on therapeutic drug testing, we conducted a series of experiments wherein primary orthotopically implanted OS tumors are removed at early stages of growth. In this study, we observed that as early as 5 days after orthotopic injection of tumor cells, GFP labeled OS cells can be seen in the lungs, while the primary tumors are not yet palpable, indicating that highly metastatic OS cells escape orthotopically induced tumors very early during primary tumor growth in the immunocompromised NSG mouse model. These findings are similar to those previously published by our group and others; however, it’s important to note that reports can vary by laboratory and immune-deficient host mouse strain and are sometimes conflicting. This is part of what fueled our previous study in which we characterized 18 OS cell lines in our own hands^11^. To our knowledge, there are no published reports of MG63.3/GFP or Hu09-H3/GFP in NSG mice for metastasis experiments. One study of HOS-MNNG conducted in NSG mice using intratibial injection without amputation surgery found a survival time consistent with tail-vein injection of HOS-MNNG tumor cells (~ 20 days), further supporting the finding that this method of tumor cell inoculation is not a spontaneous model of metastasis^25^.

Furthermore, we have found similar median survival regardless of whether the primary tumors were resected at early (7–9 mm diameter) or late stage (15–17 mm diameter) in our mouse models (Table 1). Survival curves for each cell line are shown in Fig. 2. Although significant differences were observed, this was interpreted to primarily result from improved survival for mice undergoing limb amputation at the earliest time point (black curve, day 5–7). Differences in survival for the remaining three timepoints were interpreted as unlikely to be clinically relevant with the exception of mice bearing Hu09-H3 xenografts amputated at timepoint 2 (red line, day 15) which bear a survival curve that more clearly separates from those of the last two amputation time points (Fig. 2c).

In addition to being more representative of human disease, this model may represent a more humane model to study metastasis as compared to amputation models which wait until the primary tumor reaches maximum size. This finding may also suggest that cisplatin is similarly effectively against metastases at early or late stages of development. Cisplatin, much like other conventional chemotherapeutics, is known to be most effective in rapidly dividing cells and is not targeted toward specific cellular features or driver events^26^. This may be why cisplatin therapy exerts a cytotoxic effect that carries similar impact on tumor cells growing as part of a primary tumor as well as those establishing new sites of growth in distant tissues such as the lung. It is possible that additional drug studies may identify compounds that are context specific. For example, some of our previous work suggests that OS requires specific metabolic adaptations to facilitate metastasis^12^. Early targeting may be beneficial in this context underscoring the importance of a model of early metastatic progression.

Although we present a modification to improve the study of early OS metastasis, it is important to consider the study limitations. Tumor cells grow rapidly and, in the case of immune-compromised hosts, may have fewer barriers to growth than in immune-competent mice. In addition, sub-clonal heterogeneity and subsequent selection has been hypothesized to alter the biology of cancer cell lines over time^27^. Importantly, the genomic changes favored in vitro may be distinct to those that contribute to disease progression or treatment resistance in vivo. Tumor cells are also artificially introduced into the host, with no opportunity for natural tumor initiation and co-evolution of tumor with surrounding stroma and other microenvironmental components that may contribute to the development of metastases^28^ and are known to harbor immune-suppressive effects and other barriers to treatment that are present in patients^29,30^. Expression of GFP offers an easy method for detecting and quantifying tumor burden. Although we have not identified differences in median survival in NSG mice, GFP expression has been published to impact tumorigenicity in some models^31^. This may be particularly relevant in immunocompetent mice. Finally, there are drug-specific pharmacokinetic factors to consider. Plasma and tumor levels of cisplatin were not assessed in this study, thus it is unknown whether cisplatin exposures achieved herein are congruent with the human clinical experience with cisplatin therapy. Established metastases are typically resistant to single-agent cisplatin therapy, likely because cisplatin is part of the up-front multiagent MAP chemotherapy protocol that is widely considered as standard of care^32^. Results from experiments using this model do not take prior exposure to cisplatin into account when assessing response of metastatic disease to cisplatin chemotherapy, and are specific to the cisplatin responsiveness of the MG63.3 cell line.

Based on this finding, we adjusted the timing of procedures within spontaneous metastasis experiments, allowing us to evaluate therapeutic drugs’ impact solely on metastatic progression (Fig. 1). We then performed experiments to test this method using cisplatin, a key component of the multiagent chemotherapy protocol used as frontline therapy in human OS patients. Our studies showed that cisplatin appears equally and highly effective in the treatment of lung metastasis arising from the xenografted MG63.3 OS cell line, regardless of timing of limb amputation in the spontaneous model system. However, drug testing on mice that underwent an early amputation clearly demonstrates the effect on the metastasis without interference from a co-existing primary tumor. Our studies indicate that therapeutic drug testing in spontaneous models of OS metastasis should consider the timing of tumor cell injection, surgical amputation, and concurrent status of lung colonization to accurately determine a drug’s benefit to the specific components of the overall disease burden. This is particularly true when evaluating therapies that may have a mechanism of action that relates specifically to a facet of metastatic progression.

## Data Availability

The datasets generated during and/or analyzed during the current study are available from the corresponding author on reasonable request.

## Abbreviations

OS: Osteosarcoma
GFP: Green fluorescent protein
NSG: NOD *scid* gamma
